# The Impact of Covid-19 on Women’s Mental Health and Wellbeing During Pregnancy and the Perinatal Period: A Mixed-Methods Systematic Review

**DOI:** 10.1177/00469580241301521

**Published:** 2024-11-25

**Authors:** Kanamon Pankaew, Diane Carpenter, Nalinee Kerdprasong, Juntina Nawamawat, Nisa Krutchan, Samantha Brown, Jill Shawe, Jane March-McDonald

**Affiliations:** 1 Boromarajonani College of Nursing Chakriraj, Thailand; 2 University of Plymouth, UK; 3 Boromarajonani College of Nursing Sawanpracharak Nakhonsawan, Thailand; 4 University of Plymouth, UK; 5 Royal Cornwall Hospital Cornwall, UK

**Keywords:** Covid-19, pregnancy, perinatal, mental health, impact, risk, protection, systematic review, mixed-methods

## Abstract

Review Question: What is the impact of Covid-19 upon the mental health and well-being of women during pregnancy and during the perinatal period? Inclusion criteria: empirical primary research; maternal mental health and wellbeing; perinatal period; Covid-19; English or Thai language; studies from December 2019-September 2021, updated March 2024. Exclusion criteria: secondary research, commentary, grey literature. Databases searched: CINAHL, Cochrane, JBI, Medline, PsycINFO, Clinical Key and Web of Science. Studies were assessed for bias using tools aligned with study design. A convergent integrated approach was taken whereby quantitative data was combined with qualitative data, synthesised simultaneously using Braun and Clarke Six Steps to Thematical Analysis and presented as narrative. Forty-two studies were included. Overall level of methodological quality of studies was 14 rated good, 28 fair. Overarching themes: “Impact” and “Emotional Impact.” Themes: demographic impact; mental health and socio-economic factors; obstetric factors; pre-morbidity; maternity service delivery; relationships; fear and worry, grief and loss. Commonality suggested some evidence for increased risk and prevalence for perinatal mental illness to pre-pandemic levels. Risk factors: lack/perceived lack of social support; high-risk pregnancy, complex obstetric history; prior mental illness; maternity service delivery, quality and safety; fear and worry. Results confer perinatal mental illness prominent during the pandemic though many did not suggest prevalence higher than pre-pandemic levels, or directly associated. Several factors compound risk. A small number of protective factors are identified. The dynamic processes of risk and protection need to be understood within the specific context in which they operate. The authors received no financial support for the research, authorship, and/or publication of this article. The study was not registered.

**What do we know already about this topic?**
● Increased vulnerability to mental illness during pregnancy and the perinatal period
● Risk and protective factors are associated with outcomes for mental health wellbeing and illness
● Poor perinatal mental health significantly impacts many women’s lives; some evidence to suggest increase in global prevalence during Covid-19 pandemic
**How does your research contribute to the field?**
● Confers perinatal mental illness very evident globally during the pandemic with some evidence to support higher prevalence and incidence rates and direct association with pandemic
● Provides insights into Covid-19 perinatal mental health risks and protection.
● Illustrates the significance of processes of risk and protection within the specific contexts in which they occur.
**What are your research’s implications towards theory, practice, or policy?**
● Recognise the increased vulnerability and risk for poor mental health outcomes for perinatal women during a pandemic
● Greater priority to be given to promoting psychological and emotional support for perinatal women in a future pandemic

## Introduction

Following the World Health Organisation (WHO) declaration in March 2020 of a global pandemic resulting from the novel coronavirus (Covid-19) outbreak which lasted until May 2023, varying restrictions were implemented globally.^1^ Common features of the time included national lockdowns, social isolation, high morbidity and mortality and evidence of increased risk to individual and population mental health.^2^ Likelihood of reduced availability and accessibility of protective mental health resources, disruptions in maternal health services^3^ and rising global rates of mental health, suggest the impact upon mental health and wellbeing of perinatal women, their infants and families was, and is significant.

The perinatal period, the time from pregnancy up until the end of the first year after giving birth, brings increased vulnerability for women.^4^ Increased risk to mental illness, most commonly depression and anxiety, but also obsessive-compulsive disorders, post-traumatic stress disorder (PTSD), postpartum psychosis and eating disorders, are evidenced.^5^ Estimates of anxiety and depression have suggested it affects 1 in 10 women in high income countries,^6,7^ and 1 in 5 women in middle-and low-income countries.^8^ A systematic umbrella review more recently estimated that the global pooled prevalence of perinatal depression and anxiety has significantly increased during Covid-19, affecting almost 1 in 3 women.^9^ Systematic global estimates of prevalence and incidence of perinatal mental illness are poorly established though all suggest poor perinatal mental health significantly impacts many women’s lives, as well as their infants and family’s health.^6,7^

Negative impacts of perinatal mental illness include increased risk of maternal suicide, abortion, obstetric complications, including intrauterine growth disorder and preterm labour, and poor birth outcomes such as low infant weight, difficulties with infant feeding and poor mother-infant bonding which is associated with increased risks for child behavioural, emotional and cognitive problems.^5,10^ Risk factors associated with poor perinatal mental health include: a history of mental illness,^11^ preterm infant, relationship issues and intimate partner violence,^12^ past trauma,^13^ social isolation, and economic pressures.^14^ Risk factors are higher for some individuals and communities with protective factors being restricted or absent, creating mental health inequalities.^15^ Social determinants, such as unemployment, minority ethnic status, exposure to personal or institutional racism, social economic deprivation, inadequate housing^16^ and stigma^1^ are associated with increased risk.

A systematic review and meta-analysis found protective factors for post -natal depression to be skin-to skin care, breast feeding, healthy diet, multi vitamin, vitamin D, zinc supplementation,^17^ while social support, particularly from partner and friends is well recognised as being protective for perinatal mental health.^18^ Psychological wellbeing adopting a positive as opposed to a deficit approach, is increasingly recognised as a significant aspect of mental health.^19,20^ Social support, self-efficacy and preparedness have been identified as supporting the transition to motherhood, promoting psychological wellbeing.^21^

Contributing to the development of a cohesive, quality evidence base to understand the impact of Covid-19 on perinatal women’s mental health is of prime importance given its significance for a smooth transition to motherhood and mother, infant and family health. A small number of systematic reviews on perinatal mental health in the context of Covid-19 have been undertaken, predominantly focusing on prevalence of depression and anxiety and quantifiable outcomes.^9,14,22-25^ To our knowledge, no mixed methods systematic review has been undertaken and only one integrative review^26^ on prevalence and intensity of anxiety and depression, risk and protective factors within the first wave of the pandemic. Our mixed methods systematic review extends the breadth of mental health focus within an extended timeframe in addition to examining commonalities and differences of global impact of Covid-19 upon women’s perinatal mental health and wellbeing.

The aim of the study was to evidence the impact of Covid-19 upon women’s mental health and wellbeing during pregnancy and the perinatal period. The study adopted the Preferred Reporting Items for Systematic Review and Meta Analysis, 2020 guidelines.^18^ After an initial informal scoping review to confirm the topic focus, the review question was formulated using the PI(C)O framework.

The following question was asked: What is the impact of Covid-19 upon the mental health and well-being of women during pregnancy and during the perinatal period? Objectives were to:

● identify differences and commonality of Covid-19 global impact upon perinatal women’s mental health
● identify risk and protective factors for perinatal women’s mental health and wellbeing during a pandemic.

## Materials and Methods

We undertook a mixed-methods systematic review (SR) defined as combining both qualitative and quantitative findings within a single SR to address overlapping or complementary review questions. This combines the strengths of both methodological approaches, allowing for greater depth of evidence and potential health policy and clinical application while also addressing the limitations of each methodological approach.^27,28^ Inclusion criteria were empirical primary research; maternal mental health and wellbeing; perinatal period; Covid-19; English or Thai language; studies from December 2019 to September 2021, updated March 2024. The exclusion criteria were secondary research, commentary, and grey literature. Search terms are included in Table 1.

**Table 1.** Search terms and synonyms.

Search term	Synonyms
Woman/Women/Wom*	Mother; Girl; Female
Pregnancy	Pregnant; Antenatal; Pre-natal
Childbirth	Birth; Delivery; Intrapartum; Labour
Postnatal	Post delivery; Post partum; Following/after delivery; Puerperium; Puerperal; First year of life; First 6 weeks following delivery; Neonatal period
Mental (ill) health	Mental illness; Mental disorder; Stress; Anxiety; Psychosis or puerperal psychosis; Postnatal depression; Depression; Phob*
Mental wellbeing	Happy; Content; Satisfied; Pleased; Resilien*
	Mental health; Disease
Covid-19	Coronavirus; Sars-CoV-2
Culture or cultural differences	Cultur*; Faith; Tradition; Practices; Norms; Mores

CINAHL, Cochrane, JBI, Medline, PsycINFO databases were searched by the UK site; Clinical Key and Web of Science were searched by the Thai site. Initial searches were undertaken in September 2021 and last updated March 2024. Limiters included Full Text, English or Thai, peer reviewed research articles and human subjects. Expanders were to include equivalent subjects. Boolean operators were used to combine or amalgamate search terms.

One hundred twenty-three full-text references were exported to Rayyan software, a web-based application that enabled initial blind screening of the papers by title and abstract.^29^ This was undertaken independently by the 2 international sites. Two duplicates were removed and the full texts of the remaining 121 papers were reviewed by both sites where insufficient detail was available to form a decision by title or abstract alone. Blinding was removed following completion of initial screening allowing for consideration of results, discussion between the 2 sites, and confirmation of included papers. A third-party reviewer was identified to provide moderation where a decision could not be reached. Full texts of the final selected papers were read to confirm they met the required criteria by 2 members from each site. The search was documented using the Preferred Reporting Items for Systematic Reviews and Meta-analyses.^30^ Forty-two studies ultimately met the inclusion criteria and were critically appraised.

A JBI Extraction tool^31^ was adapted to allow for a comprehensive extraction process aligned to the study’s aim and objectives. The tool was piloted independently by the 2 international sites undertaking a joint data extraction comparison exercise, with minor revision to the tool following discussion. Data extraction of included studies was undertaken by 2 team members from each site and cross-checked before synthesis. Data synthesis tables included: Overview summary; risk and protective factors; social variables; sample overview; recommendations; study strengths and weaknesses.

Studies was assessed for methodological quality using Critical Appraisal Skills Programme (CASP),^32,33^ Joanna Briggs Institute (JBI),^34^ Mixed Methods Appraisal Tool (MMAT)^35^ and CEBMa Centre for Evidenced -Based Management,^36^ appraisal tools aligned to study design. Piloting of the tools was undertaken by both sites to ensure a consistent approach and check inter-rater reliability. Studies where conflicting answers occurred were resolved through reappraisal and discussion between the teams. An independent arbitrator was used where agreement could not initially be obtained. Inclusion of studies meeting the set quality criteria (an average of 70% positive attributes for each of the appraisal tools was collaboratively agreed.

A convergent integrated approach was taken in which quantitative data was combined with qualitative data and synthesised simultaneously using Braun and Clarke^37^ Six Steps to Thematical Analysis. Included papers were coded and a code table developed. A tabulated word document was developed to display the codes, initial themes and final named themes which would then be presented in narrative form. The synthesis approach was considered appropriate as both quantitative and qualitative designs had ability to answer the review question.^27,31^ Reporting bias was addressed through data extraction, with study and sample attrition tabulated within a sample overview table. All studies were graded good, fair/acceptable, or weak to determine the level of quality of overall evidence.

**Figure 1.**
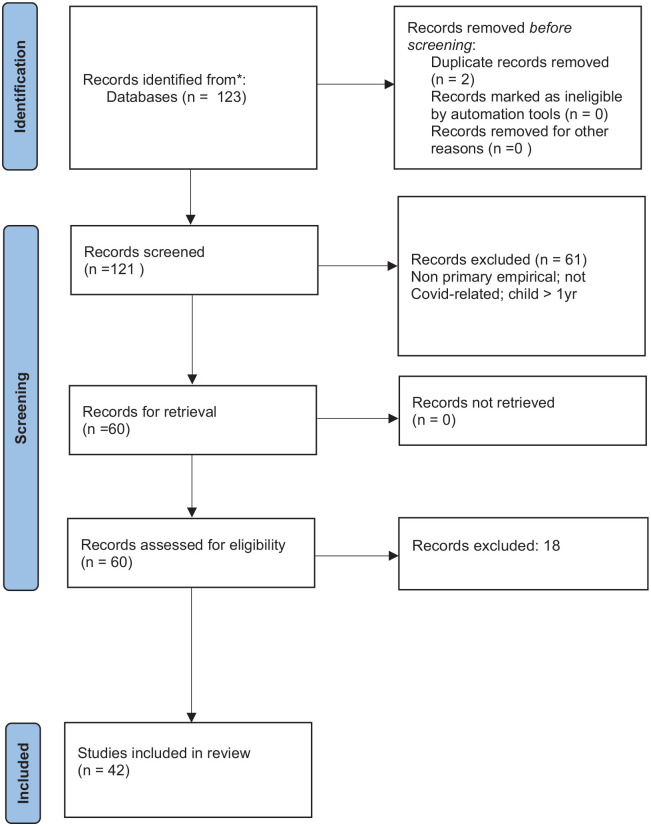
PRISMA diagram. *Source.* Page MJ, McKenzie JE, Bossuyt PM, et al. The PRISMA 2020 statement: an updated guideline for reporting systematic reviews. *BMJ*. 2021;372:n71. doi:10.1136/bmj.n71

Forty-two studies were included in the review (See Figure 1).

## Results

Seventy-nine studies were excluded as either non-primary empirical research, not Covid-related, where mothers of children outside of the perinatal period were the focus, or the studies did not reach the agreed quality status.

Included studies were comprised of 38 quantitative, 2 qualitative, and 2 mixed-methods studies. Study designs were predominantly surveys with 29 online, 7 face-to-face, 3 mixed face-to-face and online, 2 mixed online and face-to-face and 1 telephone interview; additionally, there were 32 cross-sectional studies, 2 cohort studies, 2 multi-centre cross-sectional studies, 1 longitudinal and prospective study, 1 retrospective cohort study, 2 qualitative studies, 2 mixed methods studies. Studies were conducted in 15 countries: United States of America (n = 11), Turkey (n = 7), China (n = 5), Italy (n = 4), Canada (n = 3), the United Kingdom (n = 2), Poland (n = 2), Australia (n = 1), Australia and New Zealand (n = 1), Portugal (n = 1), Spain (n = 1), Qatar (n = 1), Iran (n = 1), Israel (n = 1) and Ethiopia (n = 1). Sample size for studies adopting a quantitate approach ranged from 137.60^38^ to 72 participants,^39^ with 10 studies between 1061 and 4451 in size. A power calculation with a confidence interval of 95% was achieved in 3 studies.^40-42^ Sample sizes of studies using a qualitative approach were 27 participants^43^ and 15.^44^

All studies but two, those adopting a qualitative design^43,44^ stated use of a mental health and or risk assessment tool specific to the subject focus to assess risk, resilience, symptomology and severity, or wellbeing and aspects of functioning or development. Covid specific risk assessment tools were used in the following studies.^45-49^: Of these, all but one: Sbrilli et al^47^ were validated tools.

Thirteen studies accounted for women’s employment status, though not by occupation.^40-42,47,50-58^: Ten studies stated the sample included women with a pre-existing mental health condition, though specific numbers were not always identifiable.^45,54,55,58-63^: Mental health conditions included depression, Generalised Anxiety Disorder, anxiety disorder, Post Traumatic Stress Disorder (PTSD), mood disorder, eating disorder, obsessive compulsive disorder and parenting stress. Table 2 provides a summary of included studies.

**Table 2.** Summary of included studies.

Author, publication year and country	Target group	Methodology/study design	Face to face/online or both	Study objective	Sample size (n)	Mental health measurement/tool	Primary outcome
Ayaz R. et al. (2020), Turkey	Pregnant women	Survey	Face-to-face questionnaires	To compare the level of anxiety and depression in the same pregnant women before and during the COVID-19 pandemic.	63	- Anxiety Symptoms II (IDAS II)	COVID-19 outbreak leads to higher levels of anxiety and depression symptoms among pregnant women and affects birth outcomes.
						- Beck Anxiety Inventory (BAI)	
Chrzan-Dętkoś M. et al (2021), Poland	Pregnant women during non-epidemic and early COVID-19 epidemic period groups	Cross-sectional survey	Online questionnaires	To characterise the mental state of women in the postpartum period seeking mental health support at the beginning of the epidemic crisis in Poland.	139	- Edinburgh Postnatal Depression Scale (EPDS)	The epidemic crisis induced the severity of depressive symptoms among pregnant women compared to the pre-epidemic period. Therefore, mental health support is crucial for postpartum women.
Farrell T. et al (2020), Qatar	Pregnant woman	Cross-sectional survey in clinical setting	Face-to-face questionnaires	To study the impact of the COVID-19 pandemic and related restrictions on perinatal mental health among women in Qatar.	288	- Generalised Anxiety Disorder 7-item scale (GAD-7)	There was a high prevalence of anxiety (34.4%) and depressive symptoms (39.2%) among pregnant women during the COVID-19 pandemic.
						-PHQ-9	
Fernandes DV. et al (2021), Portugal	Mothers of babies aged between 0 and 12 months who gave birth pre- and post-COVID-19 groups	Cross-sectional online survey	Online questionnaires	1. To explore how mothers’ anxious and depressive symptoms, parenting stress, mindful parenting, and mother-infant bonding vary as a function of the moment of the baby’s birth (pre-COVID-19 or post-COVID-19). 2. To examine the contribution of those variables to mother-infant bonding.	567 Pre Covid-19 group (n = 414) and Post Covid-19 group (n = 153)	- Portuguese version of the Parental Stress Scale (PSS) - Portuguese Interpersonal Mindfulness in Parenting Scale (IM-P Infant version) - Portuguese version of Postpartum Bonding Questionnaire (PBQ)	Approximately 27.5% of the mothers presented clinically significant levels of anxious and depressive symptoms. Mothers significantly perceived difficulties in giving birth during the COVID-19 pandemic presented lower levels of emotional awareness of the child and gained more impaired mother-infant bonding than those giving birth before the pandemic period.
Fallon V. (2021), UK	Mothers with infants aged between birth and 12 weeks	Cross-sectional online survey	Online questionnaires	1. To explore the psychosocial experiences of women in the early postnatal period. 2. To describe prevalence rates of clinically relevant maternal anxiety and depression. 3. To explore whether psychosocial change occurring as a result of COVID-19 is predictive of clinically relevant maternal anxiety and depression.	614	- EPDS - State-Trait Anxiety Inventory – State scale (STAI-S) - Postpartum Specific Anxiety Scale - Research Short Form - for global - Crises (PSAS-RSF-C) - Parenting Sense of Competence scale (PSOC) - Relationship Questionnaire (RQ) - The Short Assessment of Patient Satisfaction (SAPS) - Mother-to-Infant Bonding Scale (MIBS) - COVID-19 specific items	Mothers had changed their psychosocial experiences during the social distancing period. The COVID-19 pandemic had a negative impact on mother’s mental well-being regarding the risk of clinically relevant maternal depression (30%) and anxiety (33%).
Guvenc G. et al (2020), Turkey	Postpartum woman	Cross-sectional online survey	Online questionnaires	To assess anxiety, depression, and knowledge level in postpartum women during the COVID-19 pandemic.	212	- Knowledge Assessment form regarding the COVID-19 pandemic process - State-Trait Anxiety Inventory-I (STAI-I) - EPDS	Women had higher rates of depression when their relatives tested positive for COVID-19. Women who were most anxious about COVID-19 had higher rates of depression.
Harrison V. et al (2020), UK	Pregnant woman	Online survey via digital media	Online questionnaires	To investigate the relationship between perceived social support and both anxiety and depression in UK-based pregnant women during the COVID-19 pandemic.	205	- EPDS - Perinatal Anxiety Screening Scale (PASS) - Repetitive Negative Thinking Questionnaire (RTQ-10) - Multidimensional Scale of Perceived Social Support (MSPSS) - de Jong Gierveld Loneliness Scale	Pregnant women who perceived social support tended to have less repetitive negative thinking, anxiety, loneliness, and depression.
Ilska M. et al (2021), Poland	Pregnant women in various trimesters	A cross-sectional study design	Online questionnaires	1. To create and to validate the Polish version of the original English version of the Pandemic-Related Pregnancy Stress Scale (PREPS). 2. To investigate the association of maternal obstetrical and pandemic-related factors with the PREPS to test its sensitivity.	1148	- Pandemic-Related Pregnancy Stress Scale (PREPS)	PREPS reliability measure with Cronbach’s alpha is 0.858. Pandemic-related pregnancy stress is significantly associated with fear of childbirth. Pregnant women with primiparas in the second or third trimester, receiving infertility treatment, and having a high-risk pregnancy perceived higher levels of pandemic-related stress.
Ionio C. et al (2021), Italy	Pregnant woman	Cross-sectional online survey	Online questionnaires	To explore the impact of COVID-19 on the psychic well-being of 2 samples of pregnant women (Covid epi-centre and outside epi-centre).	75	- Impact of Event Scale-Revised - Centrality of Event Scale - EPDS - Resilience scale for adults	Women who had higher perinatal depressive symptoms perceived the COVID-19 pandemic as a significant event that had low capability to plan. Residents who lived in the epicentre of Covid had a higher death rate (53.3%) and a mortality rate rising by 568% from the previous 4 years. The COVID-19 lockdown, social distancing, and fear of vital infection to the babies could lead to several risks for pregnant women’s wellbeing.
Janevic T. et al (2021), USA	Women who gave birth at two hospitals in New York City	Cross-sectional study	Online questionnaires	To examine the impact of the COVID-19 pandemic on birth satisfaction and perceived healthcare discrimination during childbirth, and in turn, the influence of these birth experiences on postpartum health.	237	- GAD-7 - Perceived Stress Scale (PSS) - Patient Health Questionnaire (PHQ2) - Birth-related PTSD	Age marginally related to satisfaction; women aged 25 to 29 rated lower than other groups. Black and Latina women who were infected with COVID-19 and gave birth during the pandemic had higher perceived healthcare discrimination, so they had lower birth satisfaction. This group was at risk of having a report of lower exclusive breastfeeding because of having higher postpartum anxiety, stress, and depressive symptoms. Postpartum stress and birth-related PTSD were the results of experiencing one or more healthcare discrimination during the peak of the COVID-19 pandemic.
Kassaw C. et al (2020), Ethiopia	Pregnant woman	Cross-sectional study	Face-to-face questionnaires	To determine the magnitude and associated factors of General anxiety disorder among perinatal service users in Dilla University referral hospital, Dilla, Ethiopia.	178	- GAD 7	Respondents residing in rural areas and had a primary educational level, were less likely to develop general anxiety disorder (GAD). On the other hand, respondents with primigravida were 3.05 times as well and respondents with poor social support were 4.39 times more likely to witness and suffer from GAD.
Koyucu RG. (2021), Turkey	Turkish pregnant women	Online survey cross-sectional study		To determine the effect of the Covid-19 pandemic on antenatal depression in Turkish pregnant women.	497	- Edinburgh Depression Scale (EDS)	The average mean scores of EDS among Turkish pregnant women were 13.70 ± 6.22. A higher pregnancy trimester was associated with higher EDS scores. There were several concerns during the COVID-19 pandemic regarding social media and news programmes related to COVID-19 (77.3%), fear of giving birth in the hospital (63.6%), planning to restrict visitors due to COVID-19 (84.7%). There were concerns about the postpartum period regarding fear of breastfeeding the infant (37%) during the COVID-19 pandemic.
Koyucu RG. and Karaca PP. (2021), Turkey	Pregnant woman	Online survey	Online questionnaires	To evaluate the mental health of pregnant women during the early and peak stages of the Covid-19 outbreak.	729	- Depression Anxiety Stress Scale - Multidimensional Perceived Social Support Scale	Pregnant women had anxiety, depression, and stress of moderate or high severity (62.2%, 44.6% and 32.2%) respectively. Pregnant woman who lost their jobs were increasing risks of threefold anxiety, sixfold of depression, and 4.8-fold of stress. Perceiving social support is the key protective factor to prevent mental health problems.
Layton (2021), Canada	Postpartum woman	RCT with before and after study	Both online and face-to-face data collection: face-to-face mental health data collection before participating in the online intervention	To determine the impact of COVID-19 pandemic on depression, anxiety, and mother-infant bonding among women seeking treatment for postpartum depression (PPD).	603 Pre COVID-19 group (n = 305) and COVID-19 group (n = 298)	- GAD-7 - Postpartum Bounding Questionnaire (PBQ)	The COVID-19 pandemic influenced anxiety among mothers with higher scores 1.63 times on the EPDS and GAD-7 among the pre-COVID-19 group. Although mothers who sought treatment for PPD during the pandemic tended to have higher levels of symptoms of depression and anxiety, they maintained good relationships with their babies.
Lebel H. et al (2020), Canada	Pregnant woman	Online survey	Online questionnaires	1. To determine the prevalence of anxiety and depression symptoms in pregnant people during the COVID-19 pandemic. 2. To identify potential resilience factors associated with lower symptoms.	1987	- EPDS - Social Support Effectiveness Questionnaire (SSEQ)	Long-term impacts of COVID-19 on babies may related to higher levels of anxiety and depression symptoms among pregnant women. Protective factors could be increased social support and exercise which were associated with lower symptoms and thus may help mitigate long-term negative outcomes.
Li C. et al (2021), China	Pregnant woman or within 8 weeks after delivering	Cross-sectional study (online survey)	Online questionnaires	1. To determine the prevalence of depression in Chinese prenatal and postnatal women after the outbreak of COVID-19. 2. To define factors that might be the risk factors for their depression.	2200	- Patient Health Questionnaire (PHQ-9) - Insomnia Severity Index (ISI) - generalised anxiety disorder scale (GAD-7)	The prevalence of depression in prenatal and postnatal women was 35.40%. Compared with the non-depression group. Factors associated with depression were drinking, nausea and vomiting during pregnancy, pregnancy’s influence on mobility, anxiety, insomnia, and daily attention to foetal movement.
Laing P. et al (2020), China	Postpartum women	Cross-sectional study	Online questionnaires	To investigate the prevalence of postpartum depression (PPD) among women in Guangzhou, China, and to explore the related factors of PPD during the COVID-19 pandemic.	845	- The Chinese version of EPDS	The women who had mild, moderate, and severe PPD were 125 (14.8%), 91(10.8%), and 37(4.4%) respectively. Factors related to PPD during the COVID-19 pandemic include immigrant women, who were at greater risk of PPD, had lower social support perceived a higher likelihood of contracting COVID-19 during the current outbreak, and were more likely to develop PPD.
Liu CH. et al (2020), USA	Pregnant women in the second trimester and postpartum women who had given birth in the past 6 months	Cross-sectional study (online REDCAP survey)	Online questionnaires	To determine if health worries due to COVID-19 and grief from experiences of loss because of the COVID-19 pandemic would be associated with higher levels of depression, generalised anxiety, and PTSD symptoms among perinatal U.S. women.	1061	- 20-item Centre for Epidemiologic Studies-Depression Measure (CES-D) - GAD 7 - PTSD Checklist—Civilian Version (PCL-C)	-Pre-existing diagnoses of depression, generalised anxiety, PTSD, and high levels of COVID-19 grief were significantly associated with a greater likelihood of clinically significant depression, anxiety, and PTSD symptoms.
Lubián López DM. et al (2021), Spain	Pregnant women	Multicenter cross-sectional study	Online questionnaires	To examine the prevalence of depressive and anxiety symptoms and the corresponding risk factors among pregnant women during the confinement due to the COVID-19 outbreak in Spain.	514	- EPDS - State-Trait Anxiety Inventory - CDRIS-10	There were 43.4% high traits and 44.2% of state anxiety scores. Samples who had a history of psychological or psychiatric diseases, comorbid depression, and lower income, and suffered from COVID-19 had significantly higher anxiety scores. Obsessive and catastrophic thoughts were the sleep disturbances which related with lower resilience scores during the pandemic. However, there was no significant correlation between anxiety levels with the length of confinement.
Mahaffey LB. et al (2021), USA	Pregnant woman	Cross-sectional study	Online questionnaires	To examine the prevalence of Obsessive-compulsive (OC) symptoms	4451	- SCID-5 - OCI-R - NuPDQ - PREPS	OCI-R obsessing and neutralising symptoms were significantly predicted by PREPS infection (β’s = 0.17 and 0.13, respectively). Neither PREPS score predicted ordering or hoarding symptoms.
Mayopoulos GA. et al (2021), USA	Postpartum women who gave birth before and during the initial outbreak of COVID-19	Cross-sectional study	Online questionnaires	To determine the association between COVID-19 and stressful childbirth and whether acute stress in birth mediates the association between COVID-19’s presence in communities and enduring posttraumatic stress and maternal bonding problems.	1274	- Peritraumatic Distress Inventory (PDI) - Posttraumatic stress disorder related to recent childbirth (CB-PTSD) - Posttraumatic Checklist for DSM-5 (PCL-5) - Mother-to-Infant Bonding Scale (MIBS) - Maternal Attachment Inventory (MAI)	Stressful childbirth during COVID-19 was related to fear of virus exposure or newborn during a hospital stay, perceiving less social support regarding visitor restrictions, and the discrepancy between pre-pandemic birth expectations and the experience of giving birth during the pandemic. Women delivering during COVID-19 had significantly higher stress which higher acute stress was associated with more childbirth-related posttraumatic stress disorder symptoms (β = .42, P < .001) and lower level of infant bonding (β = .26, P < .001), and influencing breastfeeding problems (β = .10, P < .01).
Moyer CA. et al (2020), USA	Pregnant women	Cross-sectional study	Online questionnaires	1. To explore the impact of the COVID-19 pandemic on pregnant women’s anxiety. 2. To identify factors most strongly associated with greater changes in anxiety.	2740	- PRAS scores	women are anxious about being pregnant during the COVID-19 pandemic (mean 6.5 on a scale of 1-10, 95% CI 6.4, 6.6) but more anxious about giving birth during the COVID-19 pandemic (mean 7.6 on a scale of 1-10, 95% CI 7.5, 7.6). Women in the third trimester, having a history of or recent diagnosis of depression or anxiety, having stopped in-person prenatal care, or having used the phone for prenatal care were significantly associated with greater changes in PRAS scores. However, factors associated with smaller changes in PRAS scores included higher maternal age, higher education, getting married, and planning to give birth at home.
Oskovi-Kaplan ZA. et al (2020), Turkey	Postpartum woman	Survey	Face-to-face questionnaires	To evaluate the postpartum depression rates and maternal-infant bonding status among immediate postpartum women, whose last trimester overlapped with the lockdowns and who gave birth in a tertiary care centre that had strong hospital restrictions due to serving also for COVID-19 patients, in the capital of Turkey.	525	- EPDS	EPDS scores of women with depression were significantly higher than the no-depression women. MAI score of women with depression was significantly lower than the no depression women.
Özkan Şat S. and Yaman Sözbir Ş. (2021), Turkey	Pregnant woman	A descriptive cross-sectional study	Online questionnaires	To identify the use of mobile applications by pregnant women in receiving health information, counselling, and healthcare during the COVID-19 pandemic and their distress levels during pregnancy.	376	- Tilburg Pregnancy Distress Scale (TPDS)	Pregnant women were at a risk of high distress. There was a significant difference between the change in receiving healthcare services and viral transmission.
Pariente G. et al (2020), Israel	Post-partum women who delivered during the COVID-19 strict isolation period.	A cohort study	Face-to-face questionnaires	To assess the risk for post-partum depression among women delivering during the COVID-19 pandemic as compared to the risk among women delivering before the COVID-19 pandemic.	223	- EPDS	Giving birth during the COVID-19 pandemic had a lower risk of post-partum depression compared to women delivering before the COVID-19 pandemic (16.7% vs 31.3%, P = .002).
Preis H. et al (2020), USA	Pregnant women	Survey	Online questionnaires	To investigate which sociodemographic, medical, and situational factors are most associated with greater pandemic-related pregnancy stress among pregnant women in the U.S. and which appear to be protective.	4451	- Preparedness Stress and Perinatal Infection Stress	Protective factors could be accessibility to outdoor space, seniors, and healthy behaviour engagement. Past health history associated with higher levels of stress regarding abusive history, chronic illness, loss of income due to the COVID-19 pandemic, COVID-19 pandemic risk perception, changing of prenatal appointments, having a high risk of pregnancy, ethnicity regarding women with colour were associated with greater levels of one or both types of stress.
Provenzi L. et al (2021), Italy	Pregnant woman	Longitudinal and prospective study	Both face-to-face and online questionnaires	To assess the consequences of pandemic-related prenatal stress on infants’ regulatory capacity.	163	- State-Trait Anxiety Inventory - Maternal Postpartum Attachment Scale (MPAS)	Emotional stress and receiving partial social support during pregnancy are associated with higher levels of anxiety. Mothers with postnatal anxiety were indirectly related to the capability to take care of their babies.
Ravaldi (2020), Italy	The whole country of pregnant women	Web-based survey	Online questionnaires	To investigate the psychological impact of the pandemic and lockdown on pregnant women.	737	- COVID-ASSESS questionnaire	The “lockdown” period is associated with higher levels of anxiety among women with a previous history of anxiety. Higher levels of anxiety and PTSD symptoms were found among participants who have a previous history of anxiety or depression.
Ravaldi C. (2021), Italy	Pregnant woman	Cross-sectional online survey	Online questionnaires	To explore the psychological impact of the COVID-19 pandemic on Italian pregnant women, especially regarding concerns and birth expectations	200	- COVID-ASSESS Questionnaire	Pregnant women perceived “Joy” before the COVID-19 pandemic (63%) and “Fear” after the pandemic (17%). Childbirth concerns and women’s expectations were changed by the COVID-19 pandemic.
Sakalidis VS et al (2021), Australia and New Zealand	Postpartum woman	Cross-sectional online survey	Online questionnaires	This study investigated the effect of the pandemic on feeding choices and maternal wellbeing amongst breastfeeding mothers living in Australia and New Zealand.	364	- Infant feeding practices study questionnaire (IFPS II) - Perceived Stress Scale (PSS) - General functioning subscale (GF6+) - Hardship scale - The Mental Health Continuum-Short Form (MHC-SF) - Perinatal Anxiety Screening Scale (PASS) - Brief Infant Sleep Questionnaire (BISQ)	Breastfeeding was found 82% among participants. Women who were living in higher COVID-19 infection rate areas perceived low milk supply and higher stress. Pregnancy period and partial breastfeeding related to lower milk supply and longer pregnancy duration during the COVID-19 pandemic. This is associated with stress perception, perinatal anxiety, and lower levels of family functioning.
Sbrilli MD. Et al (2021), USA	Pregnant and postpartum women	Cross-sectional online survey	Online questionnaires	To assess participants’ demographic background and the impact of the COVID-19 outbreak on the feelings and experiences of pregnant individuals and new mothers.	199 Pregnant women (n = 133) and new mothers within 6 months postpartum (n = 66)	- Early Parenthood in COVID-19 (EPiC) - Perinatal Experiences (COPE) - Brief Symptom Inventory (BSI 18) - FFMQ	Mindfulness, intolerance of uncertainty (IU), and psychological symptoms were correlated. The effects of IU and mindfulness perception were decreased during the COVID-19 pandemic.
Sharifi-Heris Z. et al (2021), Iran	Pregnant women	Cross-sectional study online study was extracted from an under-process longitudinal cohort study	Online questionnaires	To examine the association of perceived risk towards COVID-19 viral acquisition and maternal mental distress.	392	- Perceived stress scale (PSS) - State-Trait Anxiety Inventory (STAI) - Beck depression inventory (BDI-13) - Risk perception towards COVID-19 - acquisition - Protective behaviours	The COVID-19 pandemic may influence stress among pregnant women. The COVID-19 risk perception of pregnant women was a highly significant predictor for stress anxiety, depression, and protective behaviours.
Silverman ME. et al (2020), USA	Pregnant women	Retrospective cohort	Face-to-face questionnaires	To explore the mental health consequences of COVID-19-related social restrictions on pregnant women living in low socioeconomic status.	485	- EPDS	COVID-19 restrictions caused stress. Lower socio-economic status is a protective factor. Unexpected positive effects on health and well-being
Thompson KA and MA. Bardone-Cone (2021), USA	Postpartum women and woman with no pregnancy history	Online survey (case-control)	Online questionnaires	Comparison postpartum and control women on depressive, anxiety and OCD-type symptoms, and eating disorder symptoms during the 2019 nCOV pandemic and evaluated if associations between 2019-nCOV distress and these mental health symptoms differed for postpartum compared to control women	Women age 19 to 39 given birth in last 12 months (n = 232) and woman who had no pregnancy history (control group; n = 137)	- Epidemiological Studies Depression Scale (CES-D) - Depression - Anxiety Stress Scale-21 (DASS-21) - Eating Attitudes Test – 26 (EAT-26) - Perceived Stress Scale-4 - 2019-nCOV distress	Higher OCD-type symptoms among postpartum women who were concerned about contamination and responsibility for harm. The COVID-19 pandemic influenced OCD among postpartum women regarding being aware of contamination.
Vigod SN. (2021), Canada	Postpartum women	Repeated cross-sectional study using linked health administration databases	Online questionnaires	1. To compare physician visit rates for postpartum mental illness in Ontario, Canada, during the pandemic with rates expected based on pre-pandemic patterns 2. To identify variation by provider type, clinical diagnosis, postpartum timing, parity, income, ethnicity, and region of residence	137 609	N/A	Women who newly delivered babies during COVID-19 were affected the most. Patients in the lowest income group had the smallest increase in visit rates compared with other groups. The northern regions tend to have fewer mental health specialists.
Wang J. et al (2021), China	Pregnant women	Online survey cross-sectional study	Online questionnaires	To examine insomnia and psychological factors between pregnant and lying-in women during the COVID-19 pandemic and provide theoretical support for intervention research.	2235	- ISI scores	The psychological factor is the insomnia predictor. Pregnant women who lived in high-risk areas tended to have a significantly higher incidence of insomnia than lower-risk areas. Participants who had a disease history and economic losses tended to have significantly higher insomnia scores.
Wang Y. et al (2020), China	Pregnant women with COVID-19	Longitudinal single-arm cohort study. Follow-up surveys until 3 months after giving birth or having an abortion	Both face-to-face and online questionnaires	To evaluate the long-term impact of COVID-19 in pregnancy on mothers’ psychological status and infants’ neurodevelopment to explore the association between mother/baby separation and child early development	72	- PTSD Checklist-Civilian Version (PCL C) - EPDS - Ages and Stages Questionnaires, third edition (ASQ-3) - Ages and Stages Questionnaire: Social-Emotional, second edition (ASQ:SE-2)	22.2% of pregnant patients were suffering from PTSD at 3 months after delivery or post-abortion. Although the mother-baby separation median was 35 days, 49.1% of mothers chose to separate their babies for the next 8 days after maternal quarantine. The ratio of monitoring and risk in the socio/ emotional development domain at 3 months was 22.7% and 63.6% respectively. No significant neurobehavioural development at 3 months after birth.
Wu Y. et al (2020), China	Pregnant women	Multi centre cross-sectional study using EPDS	Face-to-face questionnaires	Impact of COVID-19 on the prevalence of depressive and anxiety symptoms and corresponding risk factors among pregnant women across China	4124	- EPDS	Thoughts of self-harm positively correlated with the number of confirmed cases of COVID-19 and major life events. Risks of depressive and anxiety symptoms during the outbreak were; underweight before pregnancy, primiparous, age < 35, full-time employment, middle-income, and appropriate living space.
Mizrak Sahin B. et al (2021), Turkey	Pregnant women	Qualitative generic	Telephone interview	To understand the experiences of pregnant women during the covid 19 pandemic	15	Semi-structured interview guide	- Not understanding the seriousness and fear of the unknown. - Pandemic and the disruption of pre-natal care. - Disrupted routines and social lives. - Lack of social support in pregnant women increases anxiety. - Use of phone or internet contact can relieve stress during pregnancy.
Sweet L. et al (2021), Australia	Different ethnic backgrounds and parity	Qualitative exploratory design Phase 2 of a project	Online questionnaire for phase 1 and interviewing via Zoom or telephone for phase 2	To describe childbearing women’s experiences of becoming a mother during COVID 19 pandemic in Australia	27	Semi-structured interview guide	4 Primary themes and 10 sub-themes. Themes were: going it alone, advocating for self or others, finding a way through, and keeping safe
Claridge A. et al. (2021), USA	Postpartum woman	Mixed method	Online questionnaire and telephone interview for qualitative data collection	To understand the prevalence and correlates of prenatal depression during a pandemic event.	443	- EPDS - Open-ended questionnaires	Women who were younger, unmarried, unpaid parental leave were at higher risk for depressive symptoms. Fear and anxiety affected labour and delivery. Various emotions during the COVID-19 pandemic both positive; “joy” and “excitement,” to negative; “anxiety,” “worry,” “disappointment” and “grief and loss.”
Kinser PA. et al (2021), USA	Pregnant and post-partum women up to 6 months postdelivery	Mixed methods: Cross-sectional online observational study collecting	Online questionnaire	To evaluate the experiences of pregnant and post-partum women in the early phase of the COVID-19 pandemic.	524	-Brief Symptom Inventory-18 (BSI)	Job insecurity, family concerns, eating comfort foods, resilience/adaptability score, sleep, and use of social and news media were predictors of depressive symptoms, a“nxiety, and PTSD. Increasing resilience perception was associated with mental health status improvement.

Overall level of methodological quality of included studies was 14 studies rated good and 28 rated as fair. Quantitative designs predominately lacked sample representation and lacked consideration of statistical power. A summary of assessed study quality and rated overall level of evidence is available in the Supplemental File, Table S1. Author identified study weaknesses included limited generalisability due to lack of sample diversity, particularly absence of racial, educational and socio-economic diversity.

Data was synthesised into 2 overarching themes: “Impact” and “Emotional Impact,” with additional synthesis of risk and protective factors, and commonality of features within a global context. Table 3 presents the overarching themes, themes, and sub-themes.

**Table 3.** Overarching themes, themes and sub-themes.

Overarching themes	Theme	Sub-theme
Impact	Demographic impact	High-risk infection area
		Education
		Ethnicity
		Marital status
		Age
	Mental health impact and socio-economic factors	Employment
		Income
		Economic security
		Living space
		Social support
		Isolation
	Obstetric factors	Obstetric risk
		Perinatal stage
		Primiparity
	Pre-morbidity	History of psychotic disorder
		History of chronic illness
	Maternity service delivery	Adequacy
		Quality
		Safety
	Relationships and family networks	Support
		Separation
		Baby-mother bonding
Emotional impact	Fear and Worry	The unknown
		Childbirth
		Baby-mother separation
	Grief and loss	Social support
		Employment
		Income
		Loss management

## Narrative of themes

### Impact

#### Demographic variables

Demographic variables associated with increased incidence of depression in women were found in the following studies.^39,42,63-66^ Living in a high-risk infection area and/or with a primary level of education, increased risk for perinatal anxiety and insomnia.^39^ Women of colour^63^ and immigrant women^42^ had a higher risk of developing postpartum depression (PPD), with single women at increased risk of depressive symptoms.^64^ Two studies found younger age in women to be significant for predicting prenatal depression^64,65^ whereas a Turkish study found advanced age to be predictive.^66^ Higher educational attainment^54,67^ and being married^67^ were suggested to be protective of mental health and resilience. Religion did not feature in any included studies.

#### Perceived differences: Mental health impact and socio-economic factors

Socio economic variables impacting women’s perinatal mental health were identified in the following studies.^54,58,66-68^ Employment, income, living space and social support, either on their own or compound factors were prominent in impacting mental health, either to increase risk to mental ill health or to protect it . Perceived reasons accounting for these differences of impact are given by authors. Loss of employment or economic insecurity during Covid-19 significantly negatively impacted perinatal women’s mental health; by increasing risk of depression and anxiety, and symptomology^66,68^ or increasing stress.^67^ In contrast, a study from China^58^ found pregnant women, employed full time, of middle income and with appropriate living space had increased risk of developing mental health problems. Concerns for threat to their employment given the economic fallout, and for infection, working full time outside the home, it is suggested may account for this increased risk.

Lower income was associated with higher anxiety scores for pregnant women; financial strain was reported to be a predictive value for anxiety and depression.^54,66^ Those with very low income, under 600 euros a month, or higher incomes over 3600 euros per month, however, were found to have the highest resilience scores^54^ Low socio- economic status was also found to be protective of pregnant women’s mental health, with a decrease in mental health symptoms when covid restrictions on schools and non-essential business occurred^69^ Lower socio-economic status is suggested to cause greater resilience at times of crisis, with possible amelioration of problems resulting from lockdown having unexpected positive effects upon mental health. Three studies found income had no significant negative effect upon a sample’s mental health^42,52,70^ though the latter 2 study samples were reported to refer to mostly employed women.

A lack, or perceived lack of social support and social isolation is suggested *to* have increased risk for depressive and/or anxiety symptoms, loneliness and stress,^42,44,46,51,60,64,66,71^ including for those in unsafe romantic relationships.^64^ Loss of childcare, and household conflict increased stress,^67^ with relationship status and level of perceived security significantly correlated with experiencing depressive symptoms.^64^ Disrupted routines and social lives were suggested to have contributed to negative mental health^44^ as was grief from financial loss.^64^ Good perceived social support protected mental health, reducing symptomology, repetitive thinking, and loneliness.^51,65,66^

#### Obstetric factors impacting upon perinatal women’s health

Obstetric factors identified as increasing risk to perinatal women’s mental health, were related to 3 areas: obstetric history and risk, pregnancy/perinatal stage, and parity. An obstetric history of previous C section^54^ having received infertility treatment^48^ being underweight prior to pregnancy^58^ or having a high-risk pregnancy^48,63^ was found to increase the risk of depression and/or anxiety and symptomology. A study from Qatar^40^ found highest GAD scores for women to be in the third trimester of pregnancy or the post-natal period while a Polish study^48^ found the highest pregnancy pandemic related stress levels for women occurred in the second and third trimester. Higher levels of fear and anxiety during childbirth, and those with less available support during pregnancy and postnatally, were found to have increased risk and severity of depression and anxiety.^65^ The longer duration of pregnancy occurring during the pandemic period negatively impacted upon wellbeing scores.^72^ Primipara women had highest rates of pregnancy related stress,^48,54,58^ and higher risk of anxiety and depression.^41,44,48,54^ Conversely, a Canadian study^68^ comparing anxiety and depressive symptoms by parity revealed no differences in Edinburgh Postnatal Depression Scale (EPDS) scores or anxiety scores across the groups. A planned home birth was protective against anxiety increase.^67^

#### Impact of pre-morbidity upon perinatal women’s health

A history of mental illness or chronic physical illness was suggested in several studies to increase the risk for mental illness. Anxiety, depression, and insomnia scores were found to be significantly higher in women with prior comorbid psychotic and depressive disorders,^54,56,65^ the most important factor correlating to high levels of psychopathology during “lockdown” being previous diagnoses of anxiety and/or depression.^45,62^ A Qatar study, in contrast, suggested rates of depression were not affected by previous mental health problems or pregnancy complications.^40^ A chronic physical illness before pregnancy increased the risk for mental illness in the perinatal period in one study.^39^

#### Impact of maternity service delivery upon women’s perinatal mental health

Changes to normal expected maternity service delivery, concerns regarding adequacy and safety, and service and birth satisfaction, were significant areas of service delivery negatively impacting perinatal women’s mental health. Changes to women’s birth plan, and the postponement of prenatal care significantly increased the severity of women’s depressive^64,66^ and anxiety symptomology.^73^ A change from face to face to virtual provision increased anxiety levels for Turkish pregnant women^73^ while newly delivered mothers living in Canada in areas with the most severe lockdown restrictions experienced reduced health visits which was associated with higher levels of depression.^38^

Concern for the adequacy and safety of maternity services was associated with higher levels of depression; identified variables included: lack of pre-natal care^74^ fear of viral transmission at hospital birth and women requesting elective caesarean.^75^ Women who gave birth during the peak of the pandemic, those who were SARS-CoC-2 positive, Black, and Latina women, had lower birth satisfaction and higher perceived health care discrimination, were also associated with higher depression levels.^52^ Exposure to one or more incident of healthcare discrimination was associated with higher levels of post-partum stress and birth-related PTSD.^52^ Being able to contact health professionals easily, receiving online or telephone support, and good accessibility to services were reported in one study to provide comfort to pregnant women.^44^

#### Impact upon relationships, family networks and perinatal mental health

The impact of Covid-19 on mother and family relationships, social networks and mental health highlighted differing experiences. Positive impact was found from studies in New Zealand/Australia,^72^ Portugal^59^ and Israel^76^ and included: increased opportunity for family bonding resulting in time together and less pressures,^72^ a renewed appreciation of family time, increased shared parenting responsibilities, and time with partner^59^ and increased family cohesiveness due to working from home, or not working.^38^ In contrast, a Canadian study found parents were disproportionately negatively affected by lockdown restrictions, with decreased family support contributing to lower mental health for those living in the highest restricted areas.^38^ Turkish pregnant and post-natal women lost the traditional support of their mothers staying with them which increased their loneliness and isolation.^44^ A reduction in mental health in an Australian and New Zealand study was found to be associated with lower levels of family functioning, increased perceived stress, and perinatal anxiety.^72^

Maternal infant bonding, and breast feeding were negatively affected by the pandemic.^39,52,55,59,72,75^ Prolonged mother/baby separation^39^ increased parenting stress.^59^ Higher acute stress response to childbirth^55^ lower birth satisfaction,^52^ longer pregnancy duration during the pandemic^72^ and fear of transmission through breast feeding^75^ were associated factors. A study of new mothers found a higher acute stress response to childbirth was associated with more child-birth-related PTSD symptoms and more problems with maternal bonding and breast feeding^55^ while another^52^ study found lower birth satisfaction to be associated with poor maternal infant bonding and lower exclusive breast feeding.^52^ Canadian Mothers with PPD, seeking and receiving treatment from a Psychosocial intervention consistently maintained good relationships with their infants, being unaffected by Covid-19.^61^

### Emotional Impact

Emotional impact emerged as an overarching theme with the following themes: fear and worry, grief and loss.

#### Fear and worry

Covid specific fears were identified in several studies.^44,45,49,55,64,67,68,70,75^ Women’s fear of the unknown; not understanding the seriousness of the situation, fostered anxiety.^44^ Perceived threat to mother and baby’s life contributed to substantially elevated depression and anxiety symptoms; rates far exceeding expected normal depressive and anxiety rates seen in pregnancy as well as experienced by other groups of people during the pandemic.^39,68^ An Italian study found that while joy was the most prevalent emotion expressed by 63% of mothers before Covid-19 (n = 126), it reduced to 17% (n = 34) after the pandemic onset, with fear being most prevalent 49% (n = 98).^45^ Women in a USA study were found to have mixed emotions ranging from joy and excitement, to anxiety, worry and disappointment at the changing circumstances of the birth and post-partum period.^64^ Fear and anxiety surrounding labour and delivery^55,64,67,70,75^ and of viral transmission and separation from new-born, were prominent fears.^55,64,75^

#### Grief and loss

Pregnant women grieved for the loss of their employment and income^55,64^ and changes in job status because of the pandemic. An American study found women to hold a sense of loss for missing out on the joy of typical pregnancy and post-partum experience.^64^ Loss of social support from loved ones and restriction on visiting to meet the new infant contributed to feelings of sadness.^44,64^ In a Chinese study, women infected at the start of the pandemic with the virus in the first trimester of pregnancy aborted their babies, as did one-third of cases in the second trimester.^39^

Factors which contributed to managing worry and loss included exercise, leisure activities and use of outdoor space^54,70^ which were found to be protective of mental health, as was the ability to constantly adapt to the changing circumstances and adopt self-help strategies.^43^

Table S2: Summary of risk factors can be found in the Supplemental File.

Table S3: Summary of protective factors can be found in the Supplemental File.

Recommendations for health policy, practice and future research included:

- Greater consideration of women’s psychological and emotional support needs
- by healthcare services.^55,60,64,66,71,75^
- Early detection of mental illness and mental health problems for perinatal women^57,64,66,76^
- Consideration of the role of social media and online materials in providing social support to women^41,70^
- Clinical studies on effective promotion of maternal/infant bonding during a pandemic.^59^
- Longitudinal studies to address acute and longer-term consequences of the pandemic on maternal mental health.^46^

#### Global commonality and differences

There was some evidence of commonality of impact upon mental health of perinatal women across countries, despite the heterogeneity of focus, sample and methodologies. Most studies, both global north and south, highlighted the occurrence of poor perinatal mental health during the Covid-19 pandemic. Several studies stated increased risk and prevalence rates for mental illness, symptomology, stress levels and negative emotions compared to pre pandemic actual or expected levels, suggesting a direct relationship between Covid-19 pandemic and perinatal women’s psychopathology.^40,45,49,55,56,58,59,61,71^ The most commonly identified risk factor impacting perinatal mental health by increasing risk for depressive and/or anxiety symptoms was a lack, or perceived lack of social support, and of isolation.^42,44,46,51,60,64,66,71^ Good perceived social support was protective of mental health, reducing symptomology and or loneliness in studies from 3 countries.^51,65,66^

Frequent areas or variables where an increased risk to perinatal mental health was identified included: a high risk pregnancy or complex obstetric history,^48,54,58,63^ prior diagnosis of mental illness, particularly comorbid psychotic and depressive disorders,^45,54,56,62,65^ concerns regarding maternity service quality and safety, changes, reduction or withdrawal of expected maternity service provision,^38,64,66,73,74^ and fear and worry of viral contamination, particularly in relation to anticipated/actual hospitalisation and childbirth.^49,55,64,67,68,70,75^

Reporting bias was identified for the following: 3 studies reported sample attrition^75^: 24% (n = 153)^39^; 11.11% (n = 9) and^41^ 21.74% (n = 4). Four studies reported incomplete questionnaires^66^: 16.78 (n = 166)^68^; 10.70% (n = 238),^67^ 26% (n = 1114) and^49^ 12.5% (n = 56). One study also reported inconsistency of answers 0.5% (n = 5).^66^

## Discussion

This systematic mixed methods review has sought to bring together a diverse range of evidence and methodologies to present a narrative account of what is known of the impact of Covid-19 upon perinatal women’s mental health. The global pandemic presented a unique context for women who were perinatal during this time, with taken for granted assumptions about impacts, risk and protection requiring potential new insights as to the ways in which these might operate or be understood.

The results from our review confer that perinatal mental illness, particularly depression, anxiety and stress, was very evident during the pandemic. Many review studies though were unable to or did not go so far as to suggest that prevalence and incidence rates were higher than pre-pandemic levels, or directly associated with the pandemic. Those^25,45,49,55,56,58,59,61,71^ that did are in keeping with similar conclusions reached by Shorey et al,^23^ Hessami et al,^24^ Suwalska et al^26^ and Iyengar et al.^25^ The limited methodological quality of our included studies, sample and timing heterogeneity, diverse range of adopted assessment tools and the challenges of determining changes in prevalence and incidence given the variability of pre-existing estimates, highlight limitations and inability to draw any firm conclusions.

Demographic variables increasing risk to perinatal mental health in the content of Covid-19 pandemic, including living in a high-risk infection area, being a woman of colour, or immigrant, being single and of younger age, are confirmed findings from other studies.^25,26^

Highlighted is the prominence of fear, insecurity and increased stress impacting negatively upon mental health. While fear has been recognised as a relatively common emotion for perinatal women,^77^ Covid-19 specific fears, evidenced across several countries related to viral contamination, vertical transmission of infection between mother and infant, confusion and uncertainty of the nature and extent of risk, as well as perceived threat to actual life. Anxiety and fear of potential Covid-19 infection negatively impacting mental health was also prominent in other studies.^26,78^ Knowledge of previous human coronavirus outbreak suggested pregnant women and their foetus were at increased risk of poor pregnancy outcomes.^79^ Zaigham and Andersson’s^80^ systematic review of emerging evidence during the recent pandemic on pregnant mothers infected with SARS-CoV-2E also concluded the possibility of severe maternal morbidity, perinatal deaths and possibility of vertical transmission, though major complications were rare for most mothers. Women’s fears in the early stages of the pandemic were not without some substance.

Disruption to normal maternity service provision and professional support was associated with increased anxiety and depression. Usual services that would be expected to support women manage their fears were also widely viewed as potential sources of risk, or had been withdrawn, reduced, or delivered in unfamiliar ways. Those most at risk of obstetric poor outcomes, or with a complex obstetric history requiring the likelihood of increased professional monitoring and support throughout their pregnancy, birth and post-partum period would contend with competing tensions between risk exposure and protection of themselves, foetus and newborn infant. Likewise, women with a prior mental health illness, recognised to be at significant higher risk of high levels of psychopathology, may have been further disadvantaged by a lack of available routine screening and gaps in mental healthcare provision.^81^ A study with 11 809 participants from 12 countries found that overall, about 1 in 10 women with clinically significant symptoms of perinatal mental health symptoms were receiving mental healthcare.^81^

Social isolation and loneliness, and reduced social support related to lockdown restrictions were identified as significant risk factors increasing risk for depressive and or anxiety symptoms. Other systematic review studies support these findings. Bedaso et al’s,^82^ pre-pandemic review of the relationship between social support and mental health problems during pregnancy found significant associations with risks to depression, anxiety and self-harm. A systematic review on perinatal mental health literacy found lack of social support to be the most reported cause of post-natal depression and perinatal depression among perinatal women during Covid-19.^4^

Fear of hospitalisation, particularly childbirth, a higher acute stress response to childbirth, and or lower birth satisfaction, were factors associated with increased depression levels and negative impact upon maternal infant bonding and breast feeding. Echoing this, a Canadian study of perinatal women found concerns about giving birth and restriction of partner attending to be their main worries; worry levels were found to be higher in comparison to pre-covid levels.^83^ A systematic review found post-partum women significantly more likely to report bonding problems compared to post-partum women before the pandemic, with higher levels of depression resulting in lower attachment.^25^

Conflicting findings as to the role of income and employment on perinatal mental health were found across some studies. Lower income, financial strain, economic and employment insecurity and loss of employment were identified^54,66,68,70^ as increasing mental health problems and predictive for anxiety and depression. In contrast, very low income was also found to promote resilience^54^ with low socio-economic status found to decrease mental health symptomology.^69^ Wu et al’s^58^ study of Chinese pregnant women found those working full time in employment, of middle income and with appropriate living space had increased risk of mental health problems. Author’s considered explanations for these findings provide example of the importance of looking beyond merely identification of risk or protective factors for perinatal mental health, but rather seeking to understand the dynamic processes, salience and specific contexts in which they operate. The detrimental impact of Covid-19 on businesses and employment along with increased risk of redundancies, particularly for women, during this period have been documented.^84^ Low income and financial strain are recognised risk factors for mental health during Covid-19.^84^

In a similar vein, the contrasting impact of pandemic lockdowns, seen for some to increase isolation, parental stress, and negatively impact mental and emotional health, for others, it served to enhance family life, partner relationship, and reduce family stress as a result of working from home or being unemployed. Highlighted is the false dichotomy of viewing risk and protective factors as distinct dichotomies, when both may act to expose, increase risk, or protect. Loss of childcare and necessity to undertake home schooling are suggested to have placed a heavier burden upon some women’s lives within the home.^85^

A comparatively small number of protective factors for perinatal mental health were identified in the review compared to risks, a feature also reflected in other reviews. Good perceived or actual social support was a common finding protecting mental health, both within our review and that of other studies,^26,86^ while its role more generally in supporting the transition to motherhood and promoting psychological wellbeing is also recognised.^22^ Accessibility to health care and professional support was also identified in one study.^44^ A systematic review found social support from family and partners to be the most reported facilitator to help-seeking or mental health symptom disclosure.^4^

This paper has responded to and is aligned with this special issue journal principles of “Leave no one behind” and “Endeavour to reach the furthest behind first.” The study on women’s mental health and wellbeing during the major life event of pregnancy and the perinatal period has sought to raise recognition of the need to prioritise research into this population and to increase understanding of the impact of the pandemic upon mental health.

### Health/research recommendations

- Greater consideration given to psychological and emotional support needs of perinatal women.
- Increased sample diversity; particularly racial, educational and socio-economic diversity.
- Increasing insight into the operation and impact of risk and protective factors in a global pandemic context.

### Study limitations

Study limitations include relatively small number of included papers; predominantly of fair to acceptable overall quality of evidence; under representation of studies from the global south, and geographically limited studies from the global north; the study was not registered.

### Conclusion

Perinatal mental illness and negative emotions were prominent in women’s lives during the pandemic. Women’s vulnerability and risk to adverse mental health was contributed and influenced by a range of factors operating in ways specific to the pandemic context.

## Supplemental Material

sj-docx-1-inq-10.1177_00469580241301521 – Supplemental material for The Impact of Covid-19 on Women’s Mental Health and Wellbeing During Pregnancy and the Perinatal Period: A Mixed-Methods Systematic Review Supplemental material, sj-docx-1-inq-10.1177_00469580241301521 for The Impact of Covid-19 on Women’s Mental Health and Wellbeing During Pregnancy and the Perinatal Period: A Mixed-Methods Systematic Review by Kanamon Pankaew, Diane Carpenter, Nalinee Kerdprasong, Juntina Nawamawat, Nisa Krutchan, Samantha Brown, Jill Shawe and Jane March-McDonald in INQUIRY: The Journal of Health Care Organization, Provision, and Financing

sj-docx-2-inq-10.1177_00469580241301521 – Supplemental material for The Impact of Covid-19 on Women’s Mental Health and Wellbeing During Pregnancy and the Perinatal Period: A Mixed-Methods Systematic Review Supplemental material, sj-docx-2-inq-10.1177_00469580241301521 for The Impact of Covid-19 on Women’s Mental Health and Wellbeing During Pregnancy and the Perinatal Period: A Mixed-Methods Systematic Review by Kanamon Pankaew, Diane Carpenter, Nalinee Kerdprasong, Juntina Nawamawat, Nisa Krutchan, Samantha Brown, Jill Shawe and Jane March-McDonald in INQUIRY: The Journal of Health Care Organization, Provision, and Financing

sj-docx-3-inq-10.1177_00469580241301521 – Supplemental material for The Impact of Covid-19 on Women’s Mental Health and Wellbeing During Pregnancy and the Perinatal Period: A Mixed-Methods Systematic Review Supplemental material, sj-docx-3-inq-10.1177_00469580241301521 for The Impact of Covid-19 on Women’s Mental Health and Wellbeing During Pregnancy and the Perinatal Period: A Mixed-Methods Systematic Review by Kanamon Pankaew, Diane Carpenter, Nalinee Kerdprasong, Juntina Nawamawat, Nisa Krutchan, Samantha Brown, Jill Shawe and Jane March-McDonald in INQUIRY: The Journal of Health Care Organization, Provision, and Financing

sj-docx-4-inq-10.1177_00469580241301521 – Supplemental material for The Impact of Covid-19 on Women’s Mental Health and Wellbeing During Pregnancy and the Perinatal Period: A Mixed-Methods Systematic Review Supplemental material, sj-docx-4-inq-10.1177_00469580241301521 for The Impact of Covid-19 on Women’s Mental Health and Wellbeing During Pregnancy and the Perinatal Period: A Mixed-Methods Systematic Review by Kanamon Pankaew, Diane Carpenter, Nalinee Kerdprasong, Juntina Nawamawat, Nisa Krutchan, Samantha Brown, Jill Shawe and Jane March-McDonald in INQUIRY: The Journal of Health Care Organization, Provision, and Financing
