# Supplementary material for: The Impact of Covid-19 on Women’s Mental Health and Wellbeing During Pregnancy and the Perinatal Period: A Mixed-Methods Systematic Review
Source: Inquiry. 2024 Nov 25;61:00469580241301521. doi: 10.1177/00469580241301521 (PMC11587184; doi:10.1177/00469580241301521)
Supplement: sj-docx-1-inq-10.1177_00469580241301521 – Supplemental material for The Impact of Covid-19 on Women’s Mental Health and Wellbeing During Pregnancy and the Perinatal Period: A Mixed-Methods Systematic Review [file sj-docx-1-inq-10.1177_00469580241301521.docx]

Supplemental Material

The following tables to be included as Supplemental Material

Table S1 Summary of Assessed Study Quality and Rating of Overall Evidence

Table S2 Summary of Risk Factors

Table S3 Summary of Protective Factors
