# Supplementary material for: The Impact of Covid-19 on Women’s Mental Health and Wellbeing During Pregnancy and the Perinatal Period: A Mixed-Methods Systematic Review
Source: Inquiry. 2024 Nov 25;61:00469580241301521. doi: 10.1177/00469580241301521 (PMC11587184; doi:10.1177/00469580241301521)
Supplement: sj-docx-2-inq-10.1177_00469580241301521 – Supplemental material for The Impact of Covid-19 on Women’s Mental Health and Wellbeing During Pregnancy and the Perinatal Period: A Mixed-Methods Systematic Review [file sj-docx-2-inq-10.1177_00469580241301521.docx]

| Critiquing tool | Study Design | Max Score for Positive Attributes | Rating Scale of Positive Attributes | Rating Range of Included Studies | Evidence Level for Included Studies |
| --- | --- | --- | --- | --- | --- |
| MMAT version 18 | Mixed Method | 17 | 14-17: Good  12-13: Fair  Below 12: Excluded | 13-14 | 1: Good  1: Fair  2: Excluded |
| Critical Appraisal of a Survey CEBMa (adapted) | Survey | 11 | 9-11: Good  7- 8: Fair  Below 7: Excluded | 7-10 | 10: Good  24: Fair  10: Excluded |
| CASP Qualitative Check List | Qualitative | 10 | 9-10: Good  7-8: Fair  Below 6: Excluded | 9 | 2: Good  2: Excluded |
| JBI Checklist for Prevalence Studies | Quantitative comparative study | 9 | 8-9: Good  6-7: Fair | 9 | 1: Good |
| CASP Checklist for Cohort study (adapted) | Cohort study | 9 | 8-9: Good  6-7: Fair  Below 6: Exclude | 6-7 | 3: Fair |

Table S1: Summary of assessed study quality and rated overall level of evidence
