# Supplementary material for: The Impact of Covid-19 on Women’s Mental Health and Wellbeing During Pregnancy and the Perinatal Period: A Mixed-Methods Systematic Review
Source: Inquiry. 2024 Nov 25;61:00469580241301521. doi: 10.1177/00469580241301521 (PMC11587184; doi:10.1177/00469580241301521)
Supplement: sj-docx-3-inq-10.1177_00469580241301521 – Supplemental material for The Impact of Covid-19 on Women’s Mental Health and Wellbeing During Pregnancy and the Perinatal Period: A Mixed-Methods Systematic Review [file sj-docx-3-inq-10.1177_00469580241301521.docx]

**Table 5: Summary of Risk Factors**

| **Focus** | **Author** | **Country of study** | **Risk Variable** | **MH impact** |
| --- | --- | --- | --- | --- |
| **IMPACT: Demographic Variables** | Kassaw et al (2020) | Ethiopia | Living Rural location | Anxiety & insomnia |
|  | Wang et al (2021) | China | Living High risk Infection | Anxiety & insomnia |
|  | Kassaw et al (2021)  Wang et al (2021) | Ethiopia  China | Primary Level Education | Anxiety & insomnia |
|  | Preis et al (2020)  Laing et al (2020) | USA  China | Women of colour /immigrant | PPD |
|  | Claridge et al (2021) | USA | Single | Depressive symptoms |
|  | Li et al (2021)  Claridge et al (2021) | USA  USA | Younger age | Predictive of Prenatal depression |
|  | Koyucu &Karaca (2021) | Turkey | Advanced age | Predictive of Prenatal depression |
| **IMPACT: Socio economic** | Koyucu &Karaca (2021)  Lebel et al (2020)  Moyer (2020) | Turkey  Canada  USA | -Loss of employment  -Economic insecurity | -Risk of depression and anxiety  -Increased symptomology  -Increased stress |
|  | Wu et al (2019) | China | -Pregnant  -Fulltime employment  -Middle income  -Appropriate living space | -Increased risk of mental health problems |
|  | Lubian & Lopez (2021) | Spain | Lower-income | Higher anxiety scores for pregnant women |
|  | Lubian &Lopez (2021\|)  Koyucu & Karaca (2021) | Spain  Turkey | Financial strain | Predictive for anxiety & depression |
|  | Kinser (2021)  Laing (2020)  Janevic (2021) | USA  China  USA | Income | No significant negative impact |
|  | Chrzan-Detkos et al (2021)  Fallon (2021)  Harrison (2020)  Kassaw et al(2020)  Koyucu & Karaca (2021)  Laing et al (2020)  Mizrak Sahin (2021)  Calridge et al (2021) | Poland  UK  UK  Ethiopia  Turkey  China  Turkey  USA | Lack of/perceived lack of social support /social isolation | Increased risk for depressive and/or anxiety symptoms, loneliness, stress |
|  | Claridge et al (2021) | USA | Unsafe romantic relationships | Increase depressive/anxiety symptoms |
|  | Moyer et al (2020) | USA | Loss of childcare & household stress | Increase of stress |
|  | Claridge (2021) | USA | Relationship status  & level of perceived security | Correlation with experiencing depressive symptoms |
| **IMPACT: Obstetric factors** | Lubian Lopez (2021) | Spain | Previous C section | Increased risk depression and/or anxiety and symptomology |
|  | Ilska (2021) | Poland | Having received infertility treatment | Increased risk depression and/or anxiety and symptomology |
|  | Wu et al (2020) | China | Being underweight prior to pregnancy | Increased risk depression and/or anxiety and symptomology |
|  | Preis et al (2020)  Ilska (2021) | USA  Poland | High risk pregnancy | Increased risk depression and/or anxiety and symptomology  Increased risk of stress |
|  | Farrell et al (2020) | Qatar | 3^rd^ trimester or post-natal period | Highest GAD scores |
|  | Ilska et al(2021) | Poland | 2^nd^/3^rd^ trimester | Highest pregnancy pandemic related stress levels |
|  | Li et al (2021) | China | Higher levels of fear and anxiety during childbirth | Increased risk and severity of depression and anxiety |
|  | Li et al (2021) | China | Less available support during pregnancy and postnatally | Increased risk and severity of depression and anxiety |
|  | Sakalidis et al (2021) | Australia/New Zealand | Longer duration of pregnancy during pandemic | Negatively impacted wellbeing scores |
|  | Ilska et al (2021)  Lubian Lopez et al (2021)  Wu et al (2019)  Guvenc et al (2021) | Poland  Spain  China  Turkey | Primipara | Highest rates of pregnancy related stress  Higher risk anxiety/depression |
|  | Lebel et al (2021) | Canada | Parity | No differences in anxiety or depression scores across parity groups |
| **IMPACT: Pre morbidity** | Lubian & Lopez (2021)  Oskovai-Kaplan et al (2020)  Li et al (2021) | Spain  Turkey  China | Prior comorbid psychotic and depressive disorders | Higher depression anxiety and insomnia scores |
|  | Liu et al (2020)  Ravaldi (2020) | USA  Italy | Previous diagnosis of anxiety and/or depression | The most important factors correlating to high levels of psychopathology during lockdown |
|  | Farrell et al (2020) | Qatar | Rates of depression not affected by previous MH problems or pregnancy complications | No impact on mental health |
|  | Wang J. et al (2020) | China | Chronic physical illness before pregnancy | Increased risk of mental illness/insomnia in the perinatal period |
| **IMPACT: Maternity Service delivery** | Claridge et al (2021)  Koyucu & Karaca (2021)  Ozkan Sat et al (2021) | USA  Turkey | Change to women’s birth plan | Significant increase in depressive symptomology  Increase Anxiety symptomology |
|  | Koyucu &Karaca (2021)  Ozkan Sat et al (2021) | Turkey  Turkey | Postponement of prenatal care  Change from face-to-face to virtual prenatal care | Significant increase in depressive symptomology:  Increase Anxiety symptomology |
|  | Vigod et al (2021) | Canada | Reduced health visits to newly delivered mothers during severely restricted lockdown | Higher levels of depression |
|  | Ionia et al (2021) | Italy | Lack of prenatal care during lockdown | Higher levels of depression |
|  | Korukcu et al (2022) | Turkey | Fear viral transmission at hospital birth | Higher levels of depression |
|  | Korukcu et al (2022) | Turkey | Request by women for elective caesarean section | Higher levels of depression |
|  | Janevic et al (2021) | USA | -Women who gave birth during the COVID-19 pandemic peak  -SARS-CoC-2 positive  -Black and Latino women  -Lower birth satisfaction and higher perceived health discrimination  -Exposure to 1 or more incidents of healthcare discrimination | -Higher levels of depression  -Higher post=partum stress and birth-related PTSD |
| **IMPACT: Impacts upon relationships, family networks and PMH** | Vigod et al (2021) | Canada | Decreased family support for parents in the highest restricted lockdown areas | Lower mental health |
|  | Mizrak Sahin &Kabakci (2020) | Turkey | Loss of traditional family support for pregnant and post-natal women | Increased loneliness and isolation |
|  | Wang, Y et al (2020) | China | Prolonged mother/baby separation | Associated with a negative impact on maternal-infant bonding and breastfeeding |
|  | Fernandes et al (2021) | Portugal | Mothers who gave birth during COVID-19 had Increased parenting stress | Associated with negative impact on maternal-infant bonding; lower levels of emotional awareness of the child |
|  | Mayopoulos et al (2020) | USA | Higher acute stress response to childbirth by new mothers | Associated with negative impact on maternal-infant bonding and breastfeeding problems;  More child-birth-related PTSD |
|  | Janevic et al (2021) | USA | Lower birth satisfaction | Associated with negative impact on maternal-infant bonding and breastfeeding;  Lower exclusive breastfeeding |
|  | Sakalodis et al (2021) | Australia/New Zealand | Longer pregnancy duration during the pandemic | Associated with a negative impact on maternal-infant bonding and breastfeeding |
|  | Korukcu et al (2021) | Turkey | Fear of transmission through breastfeeding | Associated with a negative impact on maternal-infant bonding and breastfeeding |
| **EMOTIONAL IMPACT:**  **Fear and Worry** | Mizrak Sahin & Kabakci (2021) | Turkey | Covid-19 specific fear of the unknown; not understanding the seriousness of the situation | Increased anxiety |
|  | Lebel et al (2020)  Sharifi-Heris et al (2021) | Canada  Iran | Worries of perceived threat of contamination and to mother & baby’s life | Substantially elevated depression and anxiety symptomology |
|  | Ravalidi et al (2021) | Italian | Increase of fear by mothers after the pandemic onset | Reduced joy from 63% to 17% |
|  | Claridge et al (2021) | USA | Changing circumstances of birth and post-partum period | Worry, anxiety and disappointment |
|  | Mayopoulos et al (2021)  Claridge et al (2021)  Moyer et al (2020)  Kinser et al (2021)  Korukcu et al (2021) | USA  USA  USA  USA  Turkey | -Labour and delivery during Covid-19  -Hospitalisation& viral transmission  -Hospitalisation& viral transmission | -Fear and anxiety |
| **EMOTIONAL IMPACT:**  **Grief and loss** | Harrison et al (2020)  Claridge et al (2021) | UK  USA | Loss of employment and income | -Grief |
|  | Claridge et al (2021) | USA | -Changes in job status due to the pandemic  -Missing out on the joy of typical pregnancy/post-partum  -Loss of social support of loved ones  -Restricted visiting | -Grief  -Sense of loss  -Sadness |
|  | Wang, Y. et al (2020) | China | Abortion | Loss, increased PTSD |
