# Supplementary material for: The Impact of Covid-19 on Women’s Mental Health and Wellbeing During Pregnancy and the Perinatal Period: A Mixed-Methods Systematic Review
Source: Inquiry. 2024 Nov 25;61:00469580241301521. doi: 10.1177/00469580241301521 (PMC11587184; doi:10.1177/00469580241301521)
Supplement: sj-docx-4-inq-10.1177_00469580241301521 – Supplemental material for The Impact of Covid-19 on Women’s Mental Health and Wellbeing During Pregnancy and the Perinatal Period: A Mixed-Methods Systematic Review [file sj-docx-4-inq-10.1177_00469580241301521.docx]

**Table S3: Summary of Protective Factors**

| **Focus** | **Author** | **Country of study** | **Protective Factor** | **MH impact** |
| --- | --- | --- | --- | --- |
| **IMPACT**  **Demographic Variables** | Lubian &Lopez  (2021)  Moyer et al (2020) | Spain  USA | Higher Educational attainment | Protective of mental health and resilience |
|  | Moyer et al (2020) | USA | Being married | Protective of MH and resilience |
| **Mental health impact and socio-economic factors** | Lubian & Lopez (2021) | Spain | Very low income under 600 euros/month OR higher income over 3600/ month | Higher resilience scores |
|  | Silverman (2020) | USA | Low socio-economic status, and covid restrictions on schools and non-essential business | Protective of women’s MH; decrease in MH symptomology |
|  | Harrison et al (2020)  Koyucu & Karaca  (2021)  Li et al (2021) | UK  Turkey  China | Good perceived social support | -Protected mental health  -Reduction in symptomology, repetitive thinking, and loneliness |
| **Obstetric Factors** | Moyer et al (2020) | USA | Planning to give birth at home | Protective against anxiety increase |
| **Maternity Service Delivery** | Sahin Mizrak et al (2021) | Turkey | -Being able to contact health professionals easily  -Receiving online/telephone support  -Good accessibility to services | -Comfort to pregnant  -women/Can relieve stress |
| **Impact upon relationships, family networks, and perinatal MH** | Sakalidis (2021) | Australia/  New Zealand | Covid-19 restrictions | Increased opportunity for family bonding, time together, less pressures |
|  | Fernandez et al (2021) | Portugal | Covid-19 restrictions | Renewed appreciation of family time increased shared parenting responsibilities. Time with partner |
|  | Pariente et al (2020) | Israel | -Covid-19 restrictions  -working from home /not working | Increased family cohesiveness |
|  | Layton et al (2021) | Canada | -Treatment-seeking mothers:  -Psychosocial intervention for mothers with PPD- | Consistently maintained good relationships with their infants, being unaffected by Covid |
|  | Lubian &Lopez  (2021)  Kinser et al (2021) | Spain  USA | -Exercise, leisure activities/  hobbies and use of outdoor space  -Exercise | -Protective of mental health and wellbeing  -Coping strategy |
